# Gas sensing performance of Ti_3_C_2_T_x_ MXene heterojunction structures in greenhouse environments: a mini review

**DOI:** 10.3389/fchem.2024.1509732

**Published:** 2024-11-27

**Authors:** Haoming Zhang, Hongyu Xu, Wen Zeng, Zhongchang Wang, Qu Zhou

**Affiliations:** ^1^ College of Engineering and Technology, Southwest University, Chongqing, China; ^2^ College of Materials Science and Engineering, Chongqing University, Chongqing, China; ^3^ Department of Quantum and Energy Materials, International Iberian Nanotechnology Laboratory (INL), Braga, Portugal

**Keywords:** Ti_3_C_2_T_x_, heterojunction, gas sensor, greenhouse, sensing mechanism

## Abstract

With the continuous advancement of smart greenhouse technologies, digital and information-based environmental monitoring has emerged as a focal point of research. The development of high-performance gas sensors is central to achieving this objective. In recent years, MXene materials have been widely applied in the field of gas sensors due to their excellent ion mobility, favorable hydrophilicity, outstanding electronic conductivity, and unique physicochemical properties. Various MXene heterojunction structures have been synthesized for gas detection. This review aims to summarize the current state of research on Ti_3_C_2_T_x_-based gas sensors, explore methods for synthesizing different morphologies of Ti_3_C_2_T_x_ heterojunction structures, and evaluate the sensing behaviors of these configurations to fully harness their potential for gas monitoring in greenhouse environments. Additionally, an in-depth analysis of the sensing mechanisms associated with Ti_3_C_2_T_x_ heterojunction structures will be provided, offering theoretical support for future investigations. The findings indicate that Ti_3_C_2_T_x_-based nanomaterials demonstrate considerable promise as high-performance sensors for gas detection in greenhouse settings. This innovative research not only provides new insights into the development of gas sensor technologies but also serves as an important foundation for the digitization of environmental monitoring.

## 1 Introduction

In recent years, greenhouses have become essential facilities for agricultural production; however, their relatively enclosed environments lead to the accumulation of toxic and harmful gases released from improper fertilizer application and insufficient ventilation (Assimakopoulos et al., 2024; Xu et al., 2024). This phenomenon has exacerbated air quality issues within greenhouses. For instance, when ammonia (NH_3_) concentration exceeds 5 mg/m^3^ (approximately 6.579 ppm), crop leaves may exhibit water-soaked spots, ultimately resulting in wilting; at concentrations above 30 mg/m³ (approximately 39.474 ppm), NH_3_ binds with hemoglobin, causing symptoms such as chest tightness, cough, and respiratory difficulties, alongside conjunctival congestion and headaches (Hassan et al., 2024; Huang W. et al., 2024; Wang X. et al., 2024). Similarly, when nitrogen dioxide (NO_2_) concentration surpasses 5 mg/m^3^ (approximately 2.433 ppm), water-soaked spots appear on the edges of crop leaves, which quickly fade to a bleached appearance; if concentrations exceed 20 mg/m^3^ (approximately 9.73 ppm), NO_2_ can inhibit enzyme activity in humans, affecting the nervous system and leading to chest tightness and respiratory distress, with long-term exposure potentially resulting in neurosis and chronic respiratory inflammation (Lu D. et al., 2023; Kwon et al., 2024; Zhang et al., 2024a). Furthermore, elevated levels of ethylene (C_2_H_4_), exceeding 2 mg/m^3^ (approximately 1.598 ppm), significantly impact the edges of leaves and young shoots, manifesting as chloroplast degradation that leads to yellowing or whitening of the leaves. Although ethylene exhibits low toxicity to humans, prolonged exposure may induce symptoms such as dizziness and lack of concentration; high concentrations can even cause paralysis (Zhang et al., 2020; Walubengo et al., 2022; Kormann et al., 2024). Therefore, the development of high-response and low-detection-limit sensors for real-time monitoring of toxic gases within greenhouse environments at room temperature is of critical significance. Such advancements would not only ensure healthy crop growth but also protect the health of agricultural workers, thereby supporting the progression of smart agriculture. Furthermore, it is essential to recognize that humidity levels within greenhouse settings play a pivotal role in determining sensor performance. Fluctuations in moisture can markedly influence both the sensitivity and stability of gas detection mechanisms, necessitating the design of sensors that can effectively operate under varying humidity conditions. This adaptability is crucial for ensuring accurate and reliable monitoring, as excessive moisture may lead to false readings or degradation of sensor materials. Addressing these challenges will enhance the efficacy of gas sensors in dynamic agricultural environments, ultimately contributing to improved environmental control and optimal crop management practices (Singh et al., 2024; Zhang T. et al., 2024; Zhao H. et al., 2024).

MXenes are a class of two-dimensional (2D) materials that have emerged as popular alternatives to graphene in recent years. Composed of transition metals (Sc, Ti, V, Cr, Zr, Nb, Mo, Hf, and Ta), X (C or N), and surface functional groups T (-O, -OH, and -F), MXenes are represented by the chemical formula M_n+1_X_n_T_x_, where n ranges from 1 to 3 (Li Q. et al., 2021; Xia et al., 2023; Wang Y. et al., 2024). Due to their exceptional ion mobility, favorable hydrophilicity, and outstanding electronic conductivity, MXenes have found extensive applications in sensors, catalysts, lithium-ion batteries, electromagnetic shielding, and supercapacitors (Li et al., 2023; Pang et al., 2023; Chen et al., 2024; Zhang J. et al., 2024). Zhang et al. successfully synthesized accordion-like V_2_CT_x_ MXene materials and combined them with SnS_2_ nanosheets, resulting in the development of a highly efficient MXene heterojunction gas sensor (Zhang et al., 2024d). Gas sensing results indicate that the V_2_CT_x_ material composite with SnS_2_ nanosheets exhibits an exceptional sensitivity towards NO_2_, being 581 times greater than that of intrinsic V_2_CT_x_ materials. Furthermore, at a relative humidity of 51.9%, rapid response and recovery times of 4.8 s and 4.7 s, respectively, are observed, alongside commendable selectivity. Wang et al. successfully incorporated SnS_2_ onto Nb_4_C_3_T_x_ materials through an *in situ* growth approach (Wang W. et al., 2024). This composite demonstrates a high response of 1046.6% to 50 ppm triethylamine at room temperature, with a response time of 11 s. Lu et al. designed a MXene/Na_2_Ti_3_O_7_@polyaniline composite, which exhibits a response of 283% to NH_3_ at room temperature with 90% humidity and achieves a detection limit of 186% (Lu et al., 2024). Mo_2_CT_x_ nanospheres were synthesized using ultrasonic technology by Wang et al. (Wang B. et al., 2024). Gas sensing tests revealed that this material exhibits a favorable response to 5 ppm of NO_2_ at room temperature. Additionally, a sensor composed of Nb_2_CT_x_ nanosheets combined with polyaniline was fabricated, demonstrating high selectivity and sensitivity towards low-concentration NH_3_ at room temperature and 87.1% relative humidity (Wang et al., 2021). Collectively, the synthesis of MXene heterojunction structures holds promise for achieving outstanding gas sensing capabilities for toxic gases in greenhouse environments at room temperature, thereby providing robust technical support for the advancement of smart agriculture.

Ti_3_C_2_T_x_ is among the most stable and cost-effective MXene materials, with a well-established preparation methodology. Its exceptional thermal stability, high electrical conductivity, and outstanding chemical and physical properties position it as a potential candidate for climate monitoring applications (Chen et al., 2021; Li W. et al., 2021; Qi et al., 2021). A two-dimensional α-Fe_2_O_3_/Ti_3_C_2_T_x_ MXene composite was designed by Zhang et al. (Zhang D. et al., 2024), and gas sensing tests demonstrated that this material exhibits a response of 0.27–100 ppm H_2_S at room temperature, with an exceptionally low detection limit. A unique sacrificial technique was employed by Qiu et al. to successfully fabricate Pd/Cu-modified Ti_3_C_2_T_x_ materials, which demonstrate a response time of 4 s to 0.5% H_2_ at room temperature, with a detection limit of 0.1% H_2_ (Qiu et al., 2024). Li et al. successfully fabricated Fe_2_O_3_/TiO_2_/Ti_3_C_2_T_x_ MXene composite sensors, which respond to 100 ppm NH_3_ at room temperature with response and recovery times of 62 s and 74 s, respectively (Li et al., 2024). Additionally, a Google Scholar investigation into the keyword “nano + Ti_3_C_2_T_x_ + gas sensor” revealed a steady increase in the publication count related to this topic from 2020 to 2023, with records showing 9, 22, 59, and 64 publications annually. Although this data may not be entirely precise, an increasing body of literature indicates that gas sensors based on Ti_3_C_2_T_x_ are garnering heightened attention. Therefore, summarizing the latest advancements in Ti_3_C_2_T_x_-based gas sensors is crucial for a deeper understanding of their advantages in monitoring toxic gases within greenhouse environments. This will not only provide a foundation for future research but also establish robust theoretical support for the development of smart agriculture.

In this review, several highly cited published studies have been selected to provide a concise overview of typical Ti_3_C_2_T_x_ MXene gas sensors. The objective of this research is to summarize and compare the exceptional performance characteristics of these gas sensors. Furthermore, various methods for synthesizing Ti_3_C_2_T_x_ MXene-based materials are presented to offer valuable references for related investigations. Finally, a brief exploration of the sensing mechanisms associated with Ti_3_C_2_T_x_ MXene gas sensors is conducted. This review not only establishes a foundation for understanding the applications of Ti_3_C_2_T_x_ MXenes in the realm of gas sensing but also elucidates potential directions for future research.

## 2 Recent advances in the gas sensing performance of Ti_3_C_2_T_x_

### 2.1 Metal atom modification of Ti_3_C_2_T_x_ MXene nanomaterials

**FIGURE 1:**
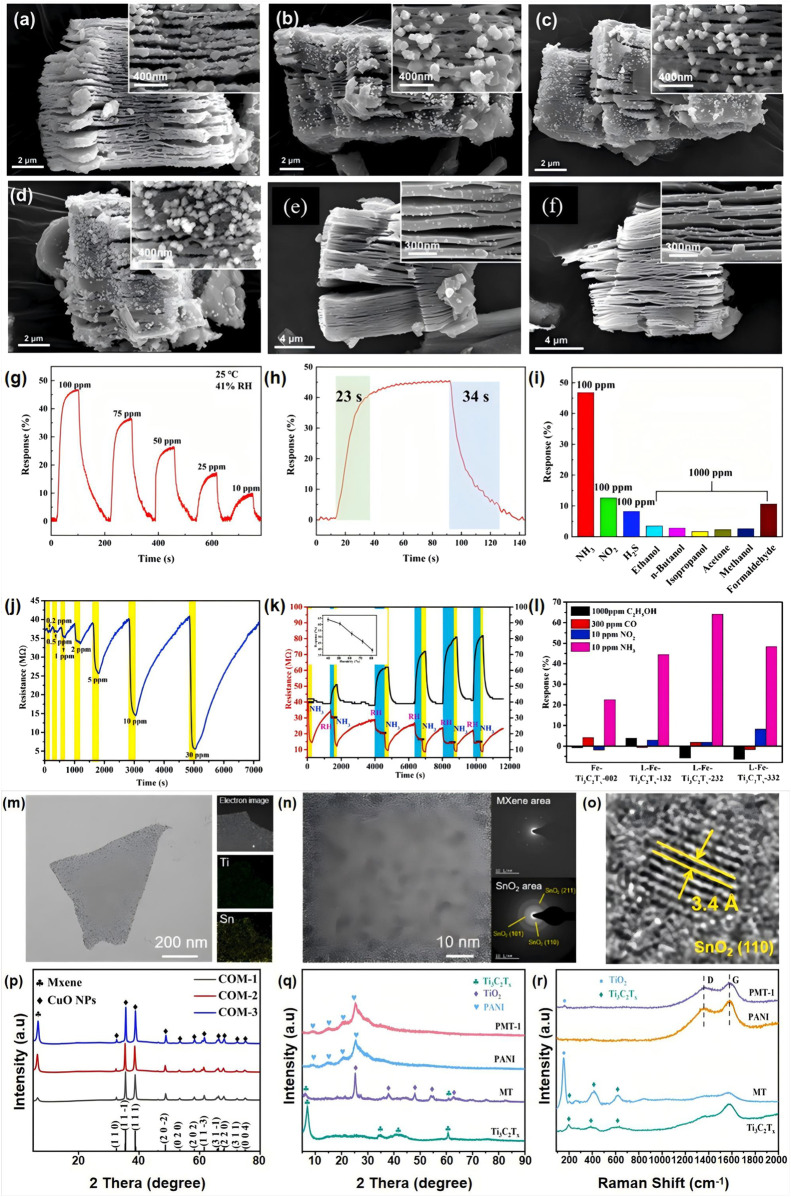
Cross-sectional SEM images of **(A)** Au (0.29 at%)- Ti_3_C_2_T_x_, **(B)** Au (1.06 at%)-Ti_3_C_2_T_x_, **(C)** Au (1.92 at%) -Ti_3_C_2_T_x_, **(D)** Au (2.48 at%)-Ti_3_C_2_T_x_ MXenes, **(E)** Pt (0.57 at%)- Ti_3_C_2_T_x_, **(F)** Pt (0.83 at%)-Ti_3_C_2_T_x_, reproduced with permission from (Nam et al., 2024). **(G)** Dynamic response curve of Pt-Ti_3_C_2_T_x_/TiO_2_ sensor at room temperature, **(H)** Typical response/recovery time of Pt-Ti_3_C_2_T_x_/TiO_2_ sensor to 100 ppm NH_3_, **(I)** Selectivity, reproduced with permission from (Zhang et al., 2023). (J) Response-recovery curve of the L-Fe-Ti_3_C_2_T_x_-232 sensor toward 0.2–30 ppm NH_3_ at RT, **(K)** Dynamic response-recovery curve of the L-Fe-Ti_3_C_2_T_x_-232 sensor toward 10 ppm NH_3_ at RT along with different RH. The inset displays the plot of the response vs. RH for 10 ppm NH_3_, **(L)** Selectivity of the sensors toward 1000 ppm ethanol, 300 ppm CO, 10 ppm NO_2_, and 10 ppm NH_3_ gas, reproduced with permission from (Qin et al., 2023). **(M)** TEM image and elemental mapping analysis of a SnO_2_-MXene hybrid nanosheet. **(N)** Magnified TEM image of SnO_2_-MXene hybrid. Insets on the right show Selected Area Electron Diffraction patterns for the exposed MXene area and SnO_2_ area. **(O)** High-Resolution Transmission Electron Microscopy image of SnO_2_ nanoparticle, reproduced with permission from (Yang et al., 2024). **(P)** XRD spectra of COM-1, COM-2, COM-3 (CuO NPs: MXene = 1:1; CuO NPs: MXene = 1:2; CuO NPs: MXene = 1:3), reproduced with permission from (Peng et al., 2024). **(Q)** XRD Patterns and **(R)** Raman spectra of Ti_3_C_2_T_x_, MT, PANI and PMT, reproduced with permission from (Xiong et al., 2023).

Nam et al. successfully developed gas sensors based on Au/Pt-modified Ti_3_C_2_T_x_ MXene (Nam et al., 2024). The Ti_3_C_2_T_x_ MXene material was synthesized through the HF etching of Ti_3_AlC_2_. The Ti_3_C_2_T_x_ MXene materials modified with varying ratios of Au/Pt particles are illustrated in Figures 1A–F. These sensors demonstrated the capability to operate in self-heating mode at room temperature, exhibiting good selectivity towards ammonia when containing 1.92 at% Au or 0.83 at% Pt NPs. Additionally, the Au-Ti_3_C_2_T_x_ MXene and Pt-Ti_3_C_2_T_x_ MXene sensors demonstrate adaptability to voltages of 5 V and 3 V, respectively, and both have successfully passed rigorous strength tests. These findings indicate that noble metal-modified Ti_3_C_2_T_x_ MXene exhibits excellent compatibility for gas detection at room temperature. This research provides novel insights into the development of high-performance gas sensors.

A method for the preparation of Pt-modified Ti_3_C_2_T_x_/TiO_2_ heterojunction sensors using a simple hydrothermal approach was proposed by Zhang et al. (Zhang et al., 2023). Experimental comparative results indicate that the response direction of the Pt-modified Ti_3_C_2_T_x_/TiO_2_ system changes significantly, enhancing its NH_3_ detection performance at room temperature compared to intrinsic Ti_3_C_2_T_x_ materials and Ti_3_C_2_T_x_/TiO_2_ heterojunctions. As shown in Figure 1G, the response of the Pt-modified Ti_3_C_2_T_x_/TiO_2_ heterojunction sensor to 100 ppm NH_3_ at room temperature is 45.5%, which is 13.8 times and 10.8 times greater than that of the intrinsic Ti_3_C_2_T_x_ material and the Ti_3_C_2_T_x_/TiO_2_ heterojunction, respectively. Furthermore, the Pt-modified Ti_3_C_2_T_x_/TiO_2_ heterojunction sensor offers advantages such as low detection limits, high response, rapid response and recovery times, and selectivity, as illustrated in Figures 1H, I. In terms of long-term stability and durability under greenhouse conditions, the Pt-Ti_3_C_2_T_x_/TiO_2_ sensor exhibited a slight decrease in response to 100 ppm NH_3_ after a duration of 22 days. This research provides new insights into the development of efficient gas sensors, particularly for monitoring harmful gases under ambient conditions.

Qin et al. reported an environmentally friendly and efficient one-step pulsed laser ablation method for synthesizing iron cluster-loaded Ti_3_C_2_T_x_ MXene (L-Fe-Ti_3_C_2_T_x_) gas sensing materials (Qin et al., 2023). Subsequently, the research team developed sensors based on L-Fe-Ti_3_C_2_T_x_ nanosheets to enable real-time detection of NH_3_ at room temperature. As illustrated in Figures 1J–L, the L-Fe-Ti_3_C_2_T_x_-232 sensor exhibited a response of 64.03% under 10 ppm NH_3_ conditions and demonstrated significant reproducibility even in high-humidity environments, making it a strong candidate for specific applications such as agricultural monitoring and human health assessment. This exceptional ammonia sensing performance is primarily attributed to the robust adsorption capability conferred by the abundant defect sites on the Ti_3_C_2_T_x_ nanosheets, along with the catalytic effects provided by the supported iron clusters. The L-Fe-Ti_3_C_2_T_x_-232 sensor demonstrated commendable long-term stability and durability under greenhouse conditions, maintaining consistent responsiveness to 10 ppm NH_3_ over a continuous monitoring period of 44 days. This study introduces a method for modifying Ti_3_C_2_T_x_ materials with non-noble metal clusters, thereby further enhancing the sensing performance of Ti_3_C_2_T_x_ MXene materials compared to previous research. Such advancements are poised to not only advance gas sensing technology but also open new avenues for practical applications in related fields.

### 2.2 Metal oxide composite Ti_3_C_2_T_x_ MXene nanomaterials

Kale et al. developed a Ti_3_C_2_T_x_ material with optimized surface terminating groups (-O and -OH), utilizing 30% HF as an etching agent to enhance the interaction with ammonia molecules, designated as Ti_3_C_2_T_x_-30 (Kale et al., 2024). This material exhibits the advantage of a low work function (3.78 eV) and is embedded in a MoO_3_ matrix to achieve both high sensitivity and room temperature operation. The MoO_3_ matrix provides stability to Ti_3_C_2_T_x_-30, effectively reducing its risk of oxidation, while Ti_3_C_2_T_x_-30 introduces additional free electrons into the MoO_3_ matrix, enabling efficient detection of NH_3_ at room temperature. When exposed to 100 ppm ammonia, the sensor response reached 93%, with a response time of approximately 10 s, representing a tenfold improvement over bare MoO_3_. The surplus electrons on the Ti_3_C_2_T_x_-30 surface facilitate the generation of species, further enhancing the stability of the MXene surface. In the presence of adsorbed MoO_3_, these species actively react with ammonia molecules, leading to significant changes in system resistance. Consequently, by incorporating an optimally proportioned Ti_3_C_2_T_x_, a significant breakthrough has been achieved for metal oxides in realizing high gas sensitivity at room temperature.

Yang et al. introduced a novel sensor by integrating SnO_2_ quantum dot (QD) nanoparticles with Ti_3_C_2_T_x_ MXene, achieving a composite material that exhibits enhanced sensing capabilities (Yang et al., 2024). The successful fabrication of this composite was thoroughly characterized through transmission electron microscopy (TEM) imaging and elemental mapping analysis, as demonstrated in Figures 1M–O. This innovative approach not only highlights the synergistic effects of combining these two materials but also paves the way for advanced gas sensing applications with improved sensitivity and selectivity. The abundant nucleation sites on the MXene surface facilitated the dense deposition of SnO_2_ QD nanoparticles with diameters ranging from 2 to 3 nm. Compared to traditional methods, SnO_2_-composited Ti_3_C_2_T_x_ MXene materials markedly improve the detection performance for NH_3_ at room temperature, exhibiting a response that is 100 times greater than that of intrinsic Ti_3_C_2_T_x_ MXene materials. These findings provide new insights into the development of gas sensors and signify the potential applications of MXene-based materials in environmental monitoring.

Peng et al. successfully synthesized transition metal oxide-derived copper oxide (CuO) nanoparticles (NPs) through a hydrothermal method combined with stirring aging techniques (Peng et al., 2024). The resultant CuO NPs exhibited octahedral growth on the surface of Ti_3_C_2_T_x_ MXene nanosheets. The successful fabrication of this composite material was substantiated by X-ray diffraction (XRD) analysis, which revealed distinct crystallographic peaks corresponding to the synthesized phases, as illustrated in Figure 1P. This innovative approach signifies a notable advancement in the integration of metal oxides with MXene substrates, paving the way for enhanced functionalities in various applications. The resulting binary heterostructure, CuO NPs/Ti_3_C_2_T_x_ MXene, retains the unique structure of the metal-organic framework (MOF), featuring abundant heterojunction interfaces. Ti_3_C_2_T_x_ MXene materials compounded with CuO nanoparticles exhibit the unique structure of metal-organic frameworks (MOFs), and the characteristics associated with MOFs result in a larger specific surface area, abundant active sites, and a more intricate porous structure, thereby significantly enhancing their adsorption performance for NO_2_. Under room temperature conditions, the sensor exhibits a sensitive response time of 38.54 s–100 ppm NO_2_, with a total response time of 2.84 s. Furthermore, the detection limit for NO_2_ is as low as 30 ppb, maintaining good stability over a testing period of 10 weeks. This research positions the composite sensor as a high-sensitivity tool for NO_2_ monitoring, showcasing significant potential for applications at room temperature.

Xiong et al. developed a flexible ammonia sensor utilizing a composite material comprising polyaniline (PANI), Ti_3_C_2_T_x_, and TiO_2_ through ultrasonic spray pyrolysis and *in situ* polymerization techniques (Xiong et al., 2023). The successful synthesis of this composite was characterized by X-ray diffraction (XRD) and Raman spectroscopy, as shown in Figures 1Q, R. This innovative methodology not only enhances the sensor’s performance but also exemplifies the potential for integrating advanced materials to facilitate heightened sensitivity and responsiveness in gas detection applications. The synthesized TiO_2_ nanoparticles are uniformly adhered to the surface of Ti_3_C_2_T_x_, effectively preventing aggregation in the PMT. Leveraging this characteristic, the enhanced PMT film sensor demonstrates high response (2.30), low detection limit (20 ppb), and high selectivity for 10 ppm NH_3_ at room temperature. Currently, considerable attention is being devoted to the incorporation of metal oxides into Ti_3_C_2_T_x_ MXene materials to form heterojunction structures. In addition to the aforementioned typical metal oxide-Ti_3_C_2_T_x_ heterojunctions, we provide an overview of representative studies on metal oxide-Ti_3_C_2_T_x_ heterojunctions for greenhouse gas detection, focusing primarily on NO_2_ and NH_3_ detection, which are summarized in Table 1.

**TABLE 1:** Summary of recent researches on Metal oxide composite Ti_3_C_2_T_x_ MXene sensors for gas detection.

Gas	Sensing material	Synthesis method	Concentration (ppm)	Temperature (°C)	Response	Selectivity	References
NH_3_	Pt-Ti_3_C_2_T_x_/TiO_2_	hydrothermal	100	room temperature	45.5%	NO_2_, H_2_S, C_2_H_6_O, C_4_H_10_O, C_3_H_8_O, C_3_H_6_O, CH_4_O, CH_2_O	Zhang et al. (2023)
	SnO_2_ QDs/Ti_3_C_2_T_x_	water-dispersible	100	room temperature	10.4	C_3_H_6_O, C_2_H_6_O, Toluene, CH_4_O	Yang et al. (2024)
	Ti_3_C_2_T_x_/CeO_2_/TiO_2_ yarns	hydrothermal	100	room temperature	42.9%	C_3_H_7_NO, CH_3_COOH, H_2_S, HCHO, C_2_H_5_OH	Zhao et al. (2024b)
	BaTiO_3_/Ti_3_C_2_T_x_	hydrothermal	20	room temperature	10.43	C_2_H_6_O, CO, SO_2_, H_2_S, C_6_H_6_	Sun et al. (2024)
	Ti_3_C_2_T_x_/SnO_2_	hydrothermal	500	room temperature	75%	C_2_H_6_OH, H_2,_ CH_4_, H_2_S, C_3_H_6_O	Yu et al. (2023)
NO_2_	Ti_3_C_2_T_x_/ZnO nanorod hybrids	hydrothermal	0.2	room temperature	346%	SO_2_, NH_3_, H_2_S, CO	Wang et al. (2022)
	In_2_O_3_/Ti_3_C_2_T_x_	hydrothermal	0.25	room temperature	193.45%	NO, CO_2_, NH_3_, CH_4_, H_2_	Fan et al. (2024)
	SnO_2_ nanoparticles@Ti_3_C_2_T_x_	one-step solvothermal	300	room temperature	78.2%	CO, H_2_, NO_2_, H_2_S, NH_3_	Liu et al. (2024)
	few-layered Ti_3_C_2_T_x_/WO_3_	electrostatic self-assembly	20	200	89.46	NH_3_, CO, C_6_H_6_, C_3_H_6_O, Xylene, Toluene, CH_4_O, CH_2_O, C_2_H_6_O	Gao et al. (2023)
	Ti_3_C_2_T_x_/ZnO	electrostatic self-assembly, hydrothermal	50	80	190%	NH_3_, C_3_H_6_O, C_7_H_8_, CH_3_OH, CH_2_O, C_3_H_8_O, C_2_H_6_O, CO	Liu et al. (2023)
C_3_H_6_O	alpha-Fe_2_O_3_/TiO_2_/Ti_3_C_2_T_x_	hydrothermal, annealing treatments	100	220	34.66	C_2_H_6_O, CH_4_O, CH_2_O, C_7_H_8_	Zhao et al. (2024c)
	α-/γ-Fe_2_O_3_/TiO_2_/Ti_3_C_2_T_x_	hydrothermal	100	240	105.5	C_8_H_10_, C_2_H_6_O, CH_4_O, CH_2_O, H_2_S, NO_2_, NH_3_, C_2_O	Huang et al. (2024b)
	TiO_2_/Fe_2_O_3_-Ti_3_C_2_T_x_	hydrothermal	100	268	446.8	C_8_H_10_, C_2_H_6_O, CH_4_O, CH_2_O, H_2_S, NO_2_, NH_3_, CO	Huang et al. (2023)
	Ti_3_C_2_T_x_/rGO/CuO	hydrothermal	100	Room Temperature	52.9%	C_6_H_6_, N(CH_3_)_3_, CH_4_O, C_7_H_8_, (C_2_H_5_)_3_N	Liu et al. (2021)
(C_2_H_5_)_3_N	In_2_O_3_ nanofibers/Ti_3_C_2_T_x_	electrospinning	50	120	12.44	CH_2_O, C_2_H_6_O, C_3_H_6_O, CH_4_O, NH_3_, C_6_H_6_	Xie et al. (2024)
	Cr_2_O_3_/TiO_2_/Ti_3_C_2_T_x_	hydrothermal	5	Room Temperature	28%	NH_3_, C_2_H_6_O, C_3_H_8_O, C_3_H_6_O, C_7_H_8_, N(CH_3_)_3_, CH_4_O, CO, NO_2_	Yao et al. (2023a)
CH_2_O	Ti_3_C_2_T_x_/SnO_2_	hydrothermal, freeze-dry	10	room temperature	29.16%	C_2_H_6_O, C_3_H_6_O, N(CH_3_)_3_, CH_4_O, NH_3_	Zhang et al. (2024a)
	ZnSnO_3_ nanocubes/Ti_3_C_2_T_x_	hydrothermal	5	room temperature	62.4%	NH_3_, C_2_H_6_O, C_6_H_6_, C_3_H_6_O, N(CH_3_)_3_	Sima et al. (2022)
C_2_H_6_O	ZnO/Ti_3_C_2_T_x_	hydrothermal	100	300	79	C_3_H_6_O, CH_4_O, C_3_H_8_O, NH_3_	Bu et al. (2023)
	MoO_2_/MoO_3_/Ti_3_C_2_T_x_	hydrothermal	200	room temperature	19.77	N(CH_3_)_3_, C_3_H_6_O, CH_4_O, CH_2_O, (C_2_H_5_)_3_N	Zhang et al. (2022)

### 2.3 Alternative materials composite Ti_3_C_2_T_x_ MXene nanomaterials

Kim et al. successfully prepared three-dimensional MoS_2_/MXene van der Waals heterostructure aerogels through the physical mixing of two-dimensional MXene and MoS_2_, followed by a freeze-drying method (Kim et al., 2023). This low-temperature synthesis approach effectively mitigated the significant oxidation of Ti_3_C_2_T_x_ MXene while forming a layered, independent three-dimensional heterostructure composed of high-quality MoS_2_ and MXene nanosheets. The catalytic layer of MoS_2_ significantly enhanced the functionalization of MXene, improving its sensitivity and long-term stability towards NO_2_ gas. Additionally, a resistive sensor based on WS_2_/Ti_3_C_2_T_x_ MXene multilayer composites was designed by Sardana et al. (Sardana et al., 2023). The optimal concentration ratio of a chemical resistive sensor was explored by combining different concentrations of WS_2_ with MXene. Notably, the composite Ti_3_C_2_T_x_ MXene sensor containing 25 wt% WS_2_ exhibited responses of 29% to 5 ppm NH_3_ and 44% to 100 ppb NO_2_ at room temperature, along with strong selectivity.

To achieve low detection limits and high sensitivity for NH_3_ detection at room temperature, a gas sensor based on a polypyrrole (PPy) coated MXene/MoS_2_ nanocomposite has been developed by Lu et al. (Lu L. et al., 2023). This loosely assembled heterogeneous structure of MXene-loaded MoS_2_ was synthesized through a one-step hydrothermal technique based on electrostatic self-assembly. The MoS_2_ nanosheets uniformly wrapped around the etched MXene surface formed a highly synergistic metal-semiconductor contact, effectively generating a resistive layer capable of capturing electrons produced during NH_3_ sensing. PPy was subsequently coated onto the MXene/MoS_2_ composite surface via an *in situ* polymerization process, resulting in a self-supporting structure. The Ti_3_C_2_T_x_ MXene/MoS_2_/PPy composite material exhibits a high response to 100 ppm NH_3_ that is five times greater than that of intrinsic Ti_3_C_2_T_x_ MXene materials, while maintaining a response of 2.08 for 10 ppm NH_3_ at room temperature. The MXene/MoS_2_/PPy nanocomposite sensor also displayed excellent long-term stability, high response and recovery rates, outstanding humidity resistance, and notable selectivity towards NH_3_.

A shell-type Ti_3_C_2_T_x_@PDAC (croconaine) composite material was designed by Zhou et al. through a simple *in situ* polymerization reaction for the detection of NH_3_ at room temperature (Zhou et al., 2023). Compared to the original Ti_3_C_2_T_x_, sensors constructed from the Ti_3_C_2_T_x_-PDAC composite exhibited remarkable sensitivity, achieving a response of 2.8% ppm^-1^ with an estimated detection limit of 50 ppb. Under room temperature conditions, the Ti_3_C_2_T_x_-PDAC sensor demonstrated commendable stability and durability, maintaining effective responsiveness to both 10 ppm and 500 ppb NH_3_ over a period of 45 days. Concurrently, Quan et al. devised a fully flexible paper-based gas sensor that integrated non-metallic Ti_3_C_2_T_x_ MXene electrodes with a Ti_3_C_2_T_x_/WS_2_ sensing film, forming ohmic contacts and Schottky heterojunctions within a single gas sensing channel (Quan et al., 2023). This Ti_3_C_2_T_x_/WS_2_ composite demonstrated high conductivity, effective charge transfer capabilities, and abundant gas-sensing active sites. Under room temperature conditions, this gas sensor achieved a response of 15.2% to 1 ppm NO_2_, which is 3.2 times greater than that of an Au fingertip electrode integrated with a Ti_3_C_2_T_x_/WS_2_ sensor (4.8%) and 76.0 times greater than that of a MXene electrode integrated with a Ti_3_C_2_T_x_ sensor (0.2%). Furthermore, the design operated effectively at a detection limit of 11.0 ppb for NO_2_ gas while exhibiting outstanding stability in high-humidity environments.

A film-type sensor based on a hybrid polyaniline (PANI:PSS) was developed by Wen et al. using a unique *in situ* polymerization technique (Wen et al., 2023). This sensor exhibits high sensitivity for NH_3_ detection, yielding a favorable response to 1 ppm NH_3_. In terms of long-term stability and durability under greenhouse conditions, the PANI:PSS/Ti_3_C_2_T_x_ sensor exhibited a mere 23% decrease in responsiveness to 1 ppm NH_3_ after 30 consecutive hours of operation at room temperature. This result underscores the sensor’s commendable stability, highlighting its potential for reliable application in extended environmental monitoring scenarios. Furthermore, it demonstrates notable selectivity, stability, and mechanical properties at room temperature. Additionally, a novel MoS_2_/Ti_3_C_2_T_x_ heterostructure sensor was designed by Ta et al. using a hydrothermal method (Ta et al., 2022). Compared to traditional heterojunction sensors, the incorporation of two-dimensional metal sulfide MoS_2_ with Ti_3_C_2_T_x_ MXene significantly enhances the sensing performance, while the compatibility between MoS_2_ and Ti_3_C_2_T_x_ ensures stable sensing capabilities for NO_2_ at room temperature. Building upon the work of Ta’s team, Tian et al. incorporated a TiO_2_ system into MoS_2_/Ti_3_C_2_T_x_ materials to enhance the capacity for electron supply in the sensing material (Tian et al., 2022). Gas sensing tests revealed that, compared to intrinsic Ti_3_C_2_T_x_ MXene and MoS_2_ materials, the sensing performance of this composite material improved by 1.79 times and 2.75 times, respectively, for 100 ppm NH_3_ at room temperature, while also exhibiting a lower detection limit. The Ti_3_C_2_T_x_ MXene@TiO_2_/MoS_2_ sensor demonstrated exceptional responsiveness to 100 ppm NH_3_ at room temperature over a continuous monitoring period of 60 days, with a slight decrease in response of only 9.1%. The response value stabilized at approximately 163.3%, indicating the sensor’s robust performance and potential for long-term applications in gas detection. These findings provide new perspectives for the development of gas sensors and illustrate the broad application potential of MXenes and their composites in environmental monitoring.

## 3 Sensing mechanisms of Ti_3_C_2_T_x_ MXene heterojunction structures

Understanding the gas sensing mechanisms is crucial for the development of high-performance sensors based on Ti_3_C_2_T_x_ MXene. The adsorbed gases can be primarily classified into oxidative and reductive gases. As shown in previous analyses, Ti_3_C_2_T_x_ MXene exhibits exceptional responsiveness to toxic gases such as NO_2_ and NH_3_ commonly found in greenhouse environments (Jin et al., 2020; Seekaew et al., 2023; Cao et al., 2024). In this study, NO_2_ is selected as a representative oxidative gas and NH_3_ as a reductive gas for in-depth investigation. Research indicates that Ti_3_C_2_T_x_ MXene, due to its high conductivity, large specific surface area, favorable hydrophilicity, and abundant surface functional groups, possesses excellent adsorption capabilities toward NO_2_ (Nahirniak and Saruhan, 2022). Furthermore, existing studies have demonstrated that Ti_3_C_2_T_x_ MXene materials can also respond favorably to the reductive gas NH_3_ (Lee et al., 2017). Consequently, the traditional charge transfer theories associated with metal oxides may not be applicable to the Ti_3_C_2_T_x_ MXene materials.

### 3.1 Sensing mechanisms of Ti_3_C_2_T_x_ MXene heterojunction structures for oxidative gases

**FIGURE 2:**
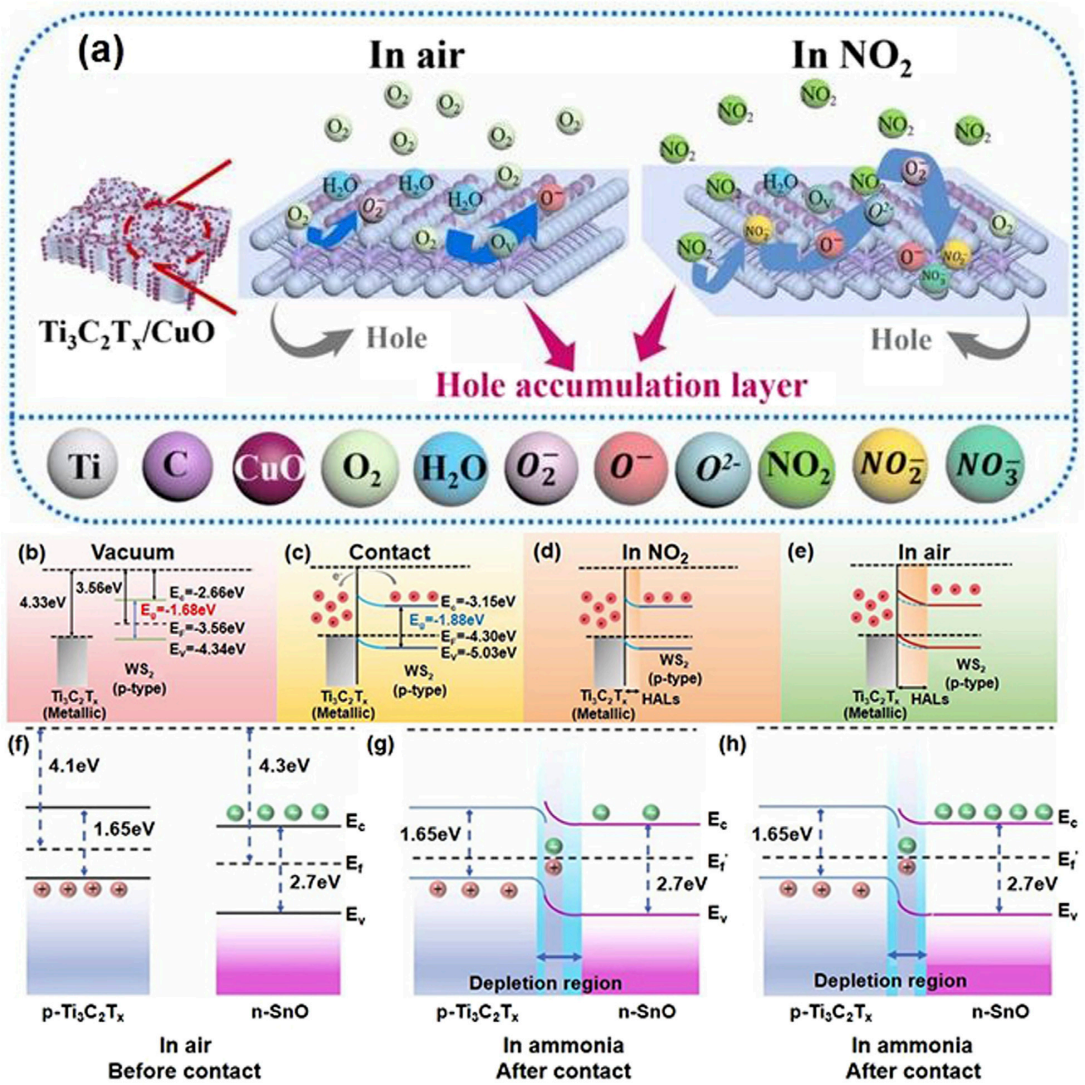
**(A)** Schematic diagrams of the gas sensing mechanism for Ti_3_C_2_T_x_/CuO sensor towards NO_2_ gas at 23°C, reproduced with permission from (Guo et al., 2023). Schematic diagrams of the work function and Fermi level position for metallic Ti_3_C_2_Tx and p-type WS_2_ semiconductor before contact **(B)**, after contact **(C)**, in the air **(D)**, and NO_2_ **(E)** at room temperature, reproduced with permission from (Quan et al., 2023). **(F–H)** the energy band diagram of Ti_3_C_2_T_x_ MXene and SnO before contact, and the p-n junction with a depletion layer at the interface between Ti_3_C_2_T_x_ MXene and SnO in air and ammonia, respectively, reproduced with permission from (Yao et al., 2022).

During the composite process of Ti_3_C_2_T_x_ MXene with In_2_O_3_, it has been observed that when NO_2_ is adsorbed at room temperature, oxygen is initially adsorbed on the surface of In_2_O_3_, where it captures electrons to form superoxide anions $O_{2}^{-}\left(\text{ads}\right)$ , as shown in Equations 1, 2 (Fan et al., 2024). Due to the greater electron affinity of NO_2_ compared to that of O_2_, electrons on the surface of the sensing material are preferentially attracted to NO_2_, resulting in the formation of ${\text{NO}}_{2}^{-}$ . The specific process is illustrated in Equations 3–5. As electrons are continually captured from In_2_O_3_ and flow towards Ti_3_C_2_T_x_, the Schottky barrier layer at the interface between Ti_3_C_2_T_x_ and In_2_O_3_ gradually expands. Upon expulsion of NO_2_ gas from the chamber, the generated ${\text{NO}}_{2}^{-}$ reacts with $O_{2}^{-}$ , reverting back to NO_2_, as illustrated in Equation 6. In parallel, Guo et al. investigated the behavior of Ti_3_C_2_T_x_/CuO sensors when exposed to air (Guo et al., 2023). Research conducted by the team reveals that the sensing mechanism of the Ti_3_C_2_T_x_/CuO sensor involves the initial interaction of oxygen molecules in the air with the sensing layer, leading to the formation of adsorbed $O_{2}\left(\text{ads}\right)$ . Subsequently, the adsorbed oxygen combines with captured electrons to form $O_{2}^{-}\left(\text{ads}\right)$ . As illustrated in Figure 2A, oxygen vacancies within the Ti_3_C_2_T_x_/CuO nanocomposite also facilitate the conversion of O_2_ (ads) into $O_{2}^{-}\left(\text{ads}\right)$ . The depletion of electrons results in a continuous increase in hole concentration within CuO. Due to the work function difference between Ti_3_C_2_T_x_ (3.9 eV) and CuO (4.7 eV), holes in CuO migrate towards Ti_3_C_2_T_x_ while electrons from Ti_3_C_2_T_x_ flow into CuO until the Fermi levels reach equilibrium. Throughout this process, the valence band thickens and the hole accumulation layer widens, resulting in an initial observation of reduced resistance in the Ti_3_C_2_T_x_/CuO sensor.

$$O_{2}\left(\text{gas}\right)\to O_{2}\left(\text{ads}\right)$$ (1)

$$O_{2}\left(\text{ads}\right)+e^{-}\to O_{2}^{-}\left(\text{ads}\right)$$ (2)

$${\text{NO}}_{2}\left(\text{gas}\right)\to {\text{NO}}_{2}\left(\text{ads}\right)$$ (3)

$${\text{NO}}_{2}\left(\text{ads}\right)+e^{-}\to {\text{NO}}_{2}^{-}\left(\text{ads}\right)$$ (4)

$${\text{NO}}_{2}\left(\text{ads}\right)+O_{2}^{-}\left(\text{ads}\right)+2e^{-}\to {\text{NO}}_{2}^{-}\left(\text{ads}\right)+2O^{-}\left(\text{ads}\right)$$ (5)

$${\text{NO}}_{2}^{-}\left(\text{ads}\right)+2O^{-}\left(\text{ads}\right)+e^{-}\to {\text{NO}}_{2}\left(\text{ads}\right)+2O_{2}^{-}\left(\text{ads}\right)$$ (6)

The MoS_2_/Ti_3_C_2_T_x_ sensor developed by Ta captures O_2_ molecules from the air at room temperature, resulting in the formation of superoxide anions as electrons are captured on the sensor surface (Ta et al., 2022). Moreover, the presence of TiO_2_ facilitates the acquisition of additional oxygen ions by the sensor. As shown in Equation 7, due to its strong electron affinity and oxidative nature, NO_2_ is capable of extracting electrons from the surrounding medium, resulting in electron depletion. The higher electronegativity of NO_2_ compared to oxygen facilitates the reaction between NO_2_ molecules and oxygen ions, leading to the formation of nitrate ions ( ${\text{NO}}_{3}^{-}$ ). Concurrently, the accumulation of holes promotes a reduction in resistance, inducing p-type semiconductor behavior. Notably, a continuous heterojunction interface forms between vertically oriented MoS_2_ and layer-stacked Ti_3_C_2_T_x_ MXene, facilitating the accelerated migration of additional electrons. In this context, edges terminated with sulfur (S) and those terminated with molybdenum (Mo) exhibit high d-orbital electron densities, enhancing electronic interactions with NO_2_ gas. Furthermore, hydrogen bonding between NO_2_ molecules and water molecules under ambient humidity conditions also contributes to the adsorption of NO_2_. In their investigation, Quan et al. similarly observed that when Ti_3_C_2_T_x_/WS_2_ materials are exposed to ambient air at room temperature, oxygen molecules can capture electrons from the material, resulting in the formation of chemically adsorbed oxide ions ( $O_{2}\left(\text{ads}\right)$ ) on the surface (Quan et al., 2023). This interaction may lead to the establishment of a hole accumulation layer. Upon the introduction of NO_2_, which possesses a higher electron affinity (2.30 eV) compared to that of oxygen (0.44 eV), electrons migrate toward the NO_2_, facilitating the formation of nitrate ions ( ${\text{NO}}_{3}^{-}$ ). This process not only promotes electron extraction but also contributes to an increase in the hole accumulation layer, ultimately resulting in a reduction in electrical resistance. The detailed reaction mechanism is illustrated in Figures 2B–E.

$$2{\text{NO}}_{2}\left(\text{ads}\right)+O_{2}^{-}\left(\text{ads}\right)+e^{-}\to 2{\text{NO}}_{3}^{-}\left(\text{ads}\right)$$ (7)

### 3.2 Sensing mechanisms of Ti_3_C_2_T_x_ MXene heterojunction structures for reductive gases

It has been noted that in TiO_2_/Ti_3_C_2_T_x_ MXene composites, TiO_2_, as an n-type semiconductor with high electronegativity, may contain intrinsic oxygen vacancies and tends to adsorb oxygen from the atmosphere on its surface (Dogra et al., 2024). The adsorbed oxygen molecules capture electrons at the oxide surface, transforming into superoxide anions ( $O_{2}^{-}$ ), as represented in Equations 8, 9. It is important to acknowledge that competition for oxygen adsorption may occur between the semiconductor oxide and Ti_3_C_2_T_x_ within the composite. In any case, the adsorbed oxygen will convert to $O_{2}^{-}$ . As an n-type semiconductor, TiO_2_ has a work function of 5.1 eV, while the typical range of the work function for Ti_3_C_2_T_x_ lies between 3.9 eV and 4.8 eV, depending on its surface terminating groups. Assuming a work function of 3.9 eV for MXene, electrons will flow from MXene to TiO_2_, leading to the formation of a depletion layer at the TiO_2_/Ti_3_C_2_T_x_ interface. When NH_3_ interacts with the sensing surface, it can contribute electrons through two different mechanisms, as illustrated in Equations 10, 11. On one hand, NH_3_ molecules may interact with the adsorbed $O_{2}^{-}$ ions, releasing electrons in the process; on the other hand, gas molecules might also interact with ${\text{OH}}^{-}$ ions, similarly resulting in electron release. The released electrons further compensate for the charge in the depletion layer, causing an increase in the sensor resistance.

$$O_{2}\left(\text{gas}\right)\to O_{2}\left(\text{ads}\right)$$ (8)

$$O_{2}\left(\text{ads}\right)+e^{-}\to O_{2}^{-}\left(\text{ads}\right)$$ (9)

$$4{\text{NH}}_{3}+5O_{2}^{-}\left(\text{ads}\right)\to 4\text{NO}\left(g\right)+6H_{2}O+e^{-}$$ (10)

$${\text{NH}}_{3}+{\text{OH}}^{-}\to {\text{NH}}_{2}+H_{2}O+e^{-}$$ (11)

Zhu et al. discovered that at room temperature (below 100 °C), oxygen adsorbed on the surface of SnO_2_/Ti_3_C_2_T_x_ nanocomposites can ionize into superoxide anions ( $O_{2}^{-}$ ), as shown in Equation 9 (Zhu et al., 2024). The entire reaction process can be described by Equations 12, 13. Yao et al. further investigated the behavior of Ti_3_C_2_T_x_/ZnO when exposed to NH_3_(Yao L. et al., 2023). Upon adsorption of NH_3_ molecules on the Ti_3_C_2_T_x_/ZnO surface, a reaction occurs with water molecules to form ammonium ions ( ${\text{NH}}_{4}^{+}$ ). Subsequently, the interactions between the adsorbed NH_3_ and ${\text{NH}}_{4}^{+}$ can also be articulated using Equations 12, 13. The electrons released from these reactions flow back to the Ti_3_C_2_T_x_/ZnO material, where they recombine with holes in Ti_3_C_2_T_x_ MXene, resulting in a reduction of hole carriers in Ti_3_C_2_T_x_/ZnO and an increase in its resistance. Subsequently, Yao et al. delved into the adsorption mechanism of NH_3_ on Ti_3_C_2_T_x_/SnO sensors and discovered that this mechanism aligns with Equations 12, 13 (Yao et al., 2022). The presence of p-n junctions enables the composite material to function as an electron acceptor, receiving electrons from the external environment. This interaction leads to a reduction in resistance during the NH_3_ adsorption process for Ti_3_C_2_T_x_ MXene/SnO, as depicted in detail in Figures 2F–H. This insightful analysis underscores how the existence of p-n junctions facilitates effective electron transfer within the compound, resulting in enhanced sensitivity to NH_3_.

$${\text{NH}}_{3}\left(\text{gas}\right)+H_{2}O\to {\text{NH}}_{4}^{+}+{\text{OH}}^{-}$$ (12)

$${\text{NH}}_{4}^{+}+O_{2}^{-}\left(\text{ads}\right)\to \text{NO}+H_{2}O+e^{-}$$ (13)

Ti_3_C_2_T_x_ MXene exhibits high conductivity, a large specific surface area, favorable hydrophilicity, and abundant surface terminal groups, enabling effective gas adsorption. However, research on the formation of heterojunctions between Ti_3_C_2_T_x_ MXene and transition metal sulfides for the detection of oxidative and reductive gases remains insufficient. Given that Ti_3_C_2_T_x_ MXene demonstrates good adsorption capabilities for both types of gases, directly applying traditional p-type semiconductor theory to Ti_3_C_2_T_x_ MXene materials may not be entirely appropriate (Kim et al., 2018; Radhakrishnan and Rout, 2023). Therefore, it is essential to consider additional factors in the analysis of resistance changes. On one hand, Ti_3_C_2_T_x_ MXene materials do not conform to the conventional definition of semiconductors; their metallic sensing layers may continually hinder the effective transport of charge carriers (Kim et al., 2018). On the other hand, the interlayer spacing of Ti_3_C_2_T_x_ MXene could influence its tolerance, representing an intriguing direction for further exploration. Additionally, the surface functional groups of Ti_3_C_2_T_x_ MXene materials play a crucial role in the gas sensing mechanism. Hydrophilic groups such as -OH and = O can interact with n-type or p-type semiconductors to enhance the adsorption of gas molecules onto the sensing material. For instance, MXene terminated with oxygen is an ideal candidate for NH_3_ sensing due to its semiconductor-like electronic properties (Bhardwaj and Hazra, 2021). Conversely, research by Hu et al. indicates that MXene terminated with sulfur is optimal for nitrogen oxide gas sensors (Hu et al., 2022). This research direction provides a new perspective on the applications of Ti_3_C_2_T_x_ MXene in gas sensing and lays the groundwork for the optimization and design of related materials.

## 4 Conclusion

This review provides a concise overview of the current research landscape surrounding gas sensors based on Ti_3_C_2_T_x_ MXene heterojunction structures. Analytical results indicate that Ti_3_C_2_T_x_ MXene nanostructures exhibit exceptional gas-sensing performance in greenhouse environments, attributed to their high electrical conductivity, extensive specific surface area, excellent hydrophilicity, and abundant surface terminal groups. The formation of heterojunction structures through composite materials has emerged as an effective strategy for enhancing the performance of Ti_3_C_2_T_x_ MXene sensors. The incorporation of metal atom doping, metal oxides, and transition metal sulfides into Ti_3_C_2_T_x_ MXene significantly improves the gas-sensing characteristics. Consequently, the development of Ti_3_C_2_T_x_ MXene heterojunction-based gas sensors presents immense potential for achieving room-temperature operation, low detection limits, and high responsiveness, particularly for gas monitoring in agricultural greenhouses. However, existing studies predominantly focus on the long-term stability and durability of these sensors under actual greenhouse conditions, mainly concerning ammonia, while investigations regarding common nitrogen oxides and other agricultural waste products remain limited. Moreover, the duration of performance stability testing is often short; thus, future research should extend these analyses to ensure prolonged usability of the sensors within intelligent greenhouse environments. In summary, this review underscores the capabilities of Ti_3_C_2_T_x_ MXene heterojunction gas sensors for monitoring toxic gases in greenhouses, highlighting their primary advantage of enabling high-sensitivity detection and rapid recovery of hazardous gases at room temperature. Future endeavors could integrate the methodologies discussed herein with reported approaches facilitating room-temperature, low-power, and highly sensitive sensing techniques, paving the way for the development of online monitoring sensor arrays for toxic gases in intelligent greenhouse systems. Additionally, establishing a quantitative detection model for multi-component mixed gases using artificial intelligence algorithms may enable direct detection of various toxic gas components within agricultural greenhouse settings.
